# CIIA prevents SOD1(G93A)-induced cytotoxicity by blocking ASK1-mediated signaling

**DOI:** 10.3389/fncel.2014.00179

**Published:** 2014-06-26

**Authors:** Jae Keun Lee, Sang Gil Hwang, Jin Hee Shin, Jaekyung Shim, Eui-Ju Choi

**Affiliations:** ^1^Laboratory of Cell Death and Human Diseases, Department of Life Sciences, School of Life Sciences and Biotechnology, Korea UniversitySeoul, South Korea; ^2^Department of Health Sciences and Technology, Samsung Advanced Institute for Health Sciences and Technology, Sungkyunkwan UniversitySeoul, South Korea; ^3^Department of Molecular Biology, Sejong UniversitySeoul, South Korea

**Keywords:** ALS (amyotrophic lateral sclerosis), ROS (reactive oxygen species), ASK1 (apoptosis signal-regulating kinase 1), mitochondria, cytotoxicity

## Abstract

Amyotrophic lateral sclerosis (ALS) is an adult-onset neurodegenerative disease with higher selectivity in degeneration of motor neurons. However, the molecular mechanism by which the ALS-linked mutants of human superoxide dismutase 1 (SOD1) gene induce neurotoxicity remains obscure yet. Here, we show that depletion of CIIA expression by RNA interference (RNAi) promoted cytotoxicity caused by ALS-linked G93A mutant of the SOD1 gene. The RNAi-mediated knockdown of CIIA also enhanced the SOD1(G93A)-induced interaction between ASK1 and TRAF2 as well as ASK1 activity. Furthermore, endogenous silencing of CIIA by RNAi augmented the effects of SOD1(G93A) on reduction of mitochondria membrane potential (Δψ_m_), release of cytochrome c into the cytoplasm, and caspase activation. Together, our results suggest that CIIA negatively modulates ASK1-mediated cytotoxic signaling processes in a SOD1(G93A)-expressing cellular model of ALS.

## Introduction

Amyotrophic lateral sclerosis (ALS) is an adult-onset neurodegenerative disease with higher selectivity in degeneration of upper and lower motor neurons. The majority of ALS are sporadic, while only 10% of the total ALS are familial. Both the sporadic and familial cases share similar clinical characteristics through possible common mechanisms of the disease. Mutation in the gene encoding superoxide dismnutase-1 (SOD1) is the primary genetic cause of familial ALS (fALS) and provokes the disease with acquired toxic gain-of-function (Gurney et al., 1994; Cleveland and Rothstein, 2001). A number of mechanisms have been proposed to elicit degenerative courses of motor neurons in ALS (Cleveland and Rothstein, 2001; Pasinelli and Brown, 2006). However, the molecular processes underlying the neurotoxicity induced by SOD1 mutants are not understood completely yet. It was previously reported that apoptosis signal-regulating kinase 1 (ASK1) may mediate ALS-associated neurotoxicity with genetic ablation of *ASK1* alleviating fALS disease features in SOD1 mutant transgenic mice (Nishitoh et al., 2008). ASK1 was activated in motor neurons in the lumbar spinal cord of SOD1(G93A) mice along with disease progression (Wengenack et al., 2004; Holasek et al., 2005; Veglianese et al., 2006) and mediated cell-autonomous death pathways induced by SOD1(G93A) (Raoul et al., 2002; Nishitoh et al., 2008). Furthermore, ASK1 appears to mediate MST1-dependent neurotoxicity of motor neurons in ALS model mice (Lee et al., 2013).

A caspase-activated DNase inhibitor that interacts with ASK1 (CIIA) was originally discovered as an anti-apoptotic factor that protects cells from apoptosis induced by tumor necrosis factor-α and a variety of cellular stress such as oxidative stress through the inhibition of ASK1 (Cho et al., 2003; Kim et al., 2010). Given that ASK1 contributes to neurodegeneration in ALS, we investigated the possible role of CIIA in the regulation of ASK1-mediated signaling initiated by SOD1(G93A). Here, we showed that CIIA mitigates SOD1(G93A)-induced cytotoxicity with inhibiting the SOD1(G93A)-induced activation of ASK1-p38 MAPK signaling. These results suggest that CIIA functions as a negative modulator of ASK1-mediated cytotoxic signaling processes in SOD1(G93A)-expressing cellular model of ALS.

## Results and discussion

### CIIA suppresses SOD1(G93A)-induced cytotoxicity

**Figure 1:**
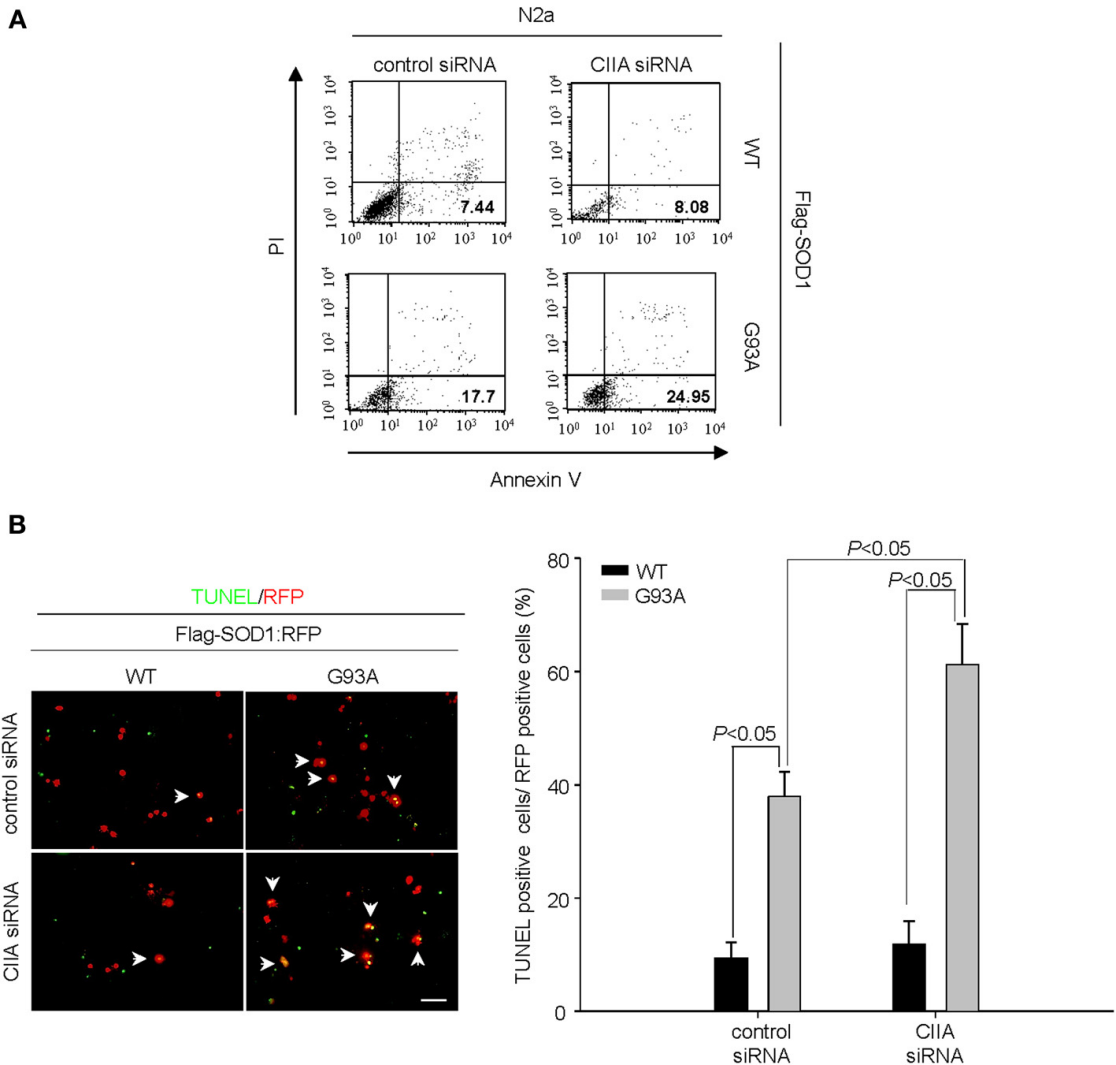
**Depletion of CIIA by RNAi potentiates SOD1(G93A)-induced cytotoxicity. (A)** Neuro2a (N2a) cells expressing Flag-tagged human SOD1(WT) or SOD1(G93A) were transfected for 48 h with si RNAs specific for GFP or CIIA mRNAs. The cells were stained with annexin V and PI, and the percentages of apoptotic cells were determined by flow cytometry. **(B)** NSC34 cells expressing Flag-tagged SOD1(WT) or SOD1(G93A) were transfected for 48 h with plasmids encoding RFP along with GFP (control) or CIIA siRNA. The cells were then examined for apoptosis by TUNEL assay. Left, representative images of the assay (scale bar, 100 μm). Arrows indicate TUNEL-positive cells. Right, the data are means ± s.e.m. from three independent experiments.

Given that CIIA has been shown to prevent apoptotic cell death induced by various cellular stresses including oxidative stress (Cho et al., 2003), we examined whether CIIA affects SOD1(G93A)-induced apoptosis. Depletion of CIIA expression by RNA interference (RNAi) enhanced SOD1(G93A)-induced apoptosis in Neuro2A (N2a) cells (Figure 1A). We further analyzed the effect of CIIA on SOD1(G93A)-induced apoptosis in NSC34 mouse motor neuron-like cells, and observed similar results (Figure 1B).

### CIIA inhibits SOD1(G93A)-induced ASK1 activity

**Figure 2:**
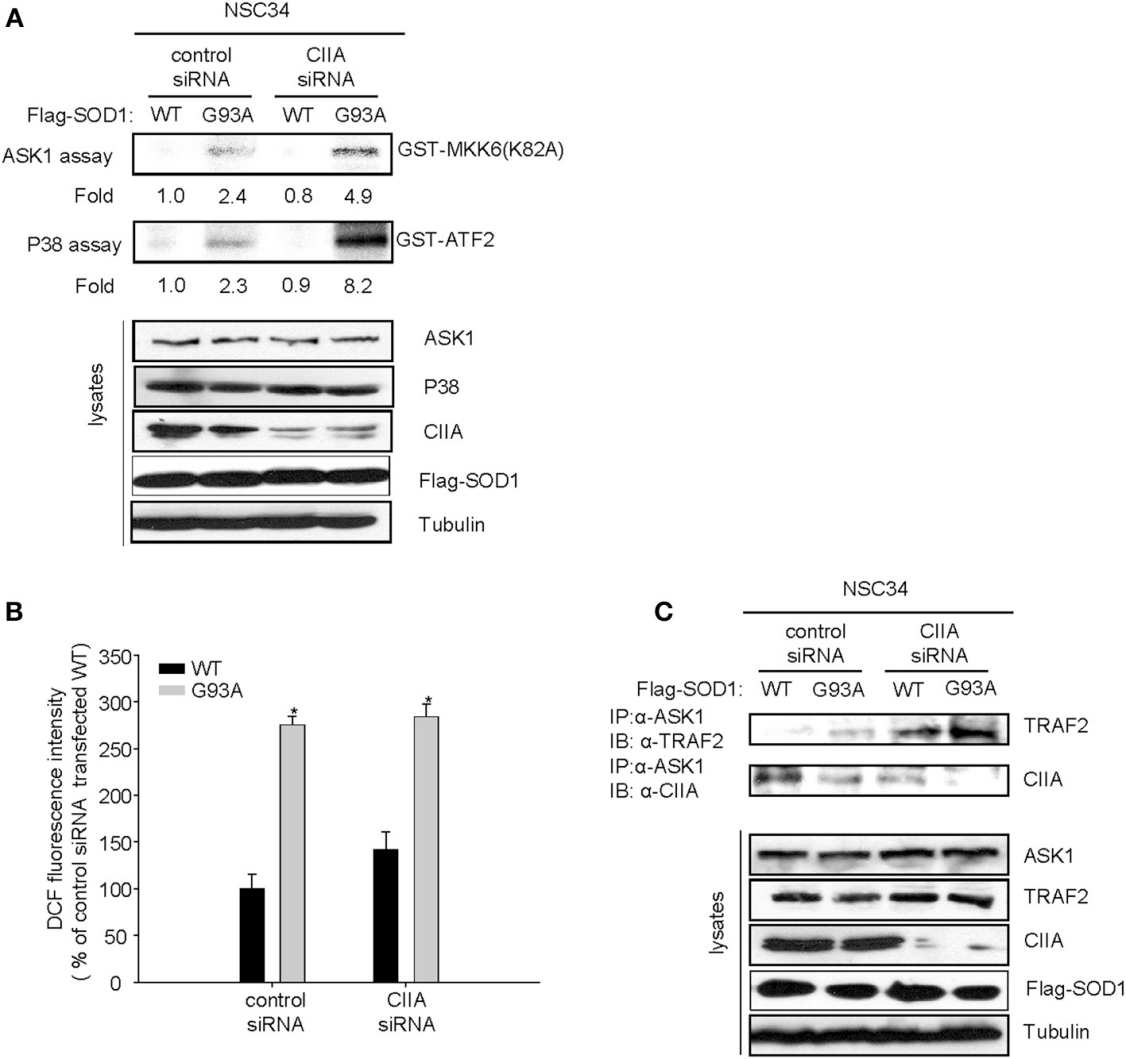
**CIIA inhibits SOD1(G93A)-induced activation of ASK1**. NSC34 cells cells were stably transfected with plasmid vectors encoding Flag-tagged SOD1(WT) or SOD1(G93A) along with vectors for GFP or CIIA siRNA. **(A,B)** Cell lysates of the stable transfectants were examined for ASK1 and p38 kinase activities by immune complex kinase assay **(A)** or for intracellular ROS production by DCF fluorescence **(B)**. The data are means ± s.e.m. from three independent experiments. ^*^P < 0.05 vs. WT. **(C)** Cell lysates were subjected to immunoprecipitation with antibodies to ASK1, and the resulting precipitates were immunoblotted with antibodies to TRAF2 or to CIIA.

ASK1, a member of the MAP3K family, has been shown to mediate SOD1(G93A)-induced neurotoxicity in an ALS mouse model (Nishitoh et al., 2008). Indeed, we observed that overexpression of ASK1(K709R), a dominant-negative mutant of ASK1, reduced SOD1(G93A)-induced apoptosis in NSC34 cells (Supplementary Figure 1), suggesting that ASK1 is associated with the mechanism of SOD1(G93A)-induced cytotoxicity. Moreover, the scavengers of reactive oxygen species (ROS) including Trolox and *N*-acetylcysteine (NAC) abrogated the SOD1(G93A)-induced increase in ASK1 activity (Supplementary Figure 2), suggesting that SOD1(G93A) induces ASK1 activation in a ROS-dependent manner. Given that CIIA was initially identified as an inhibitory protein of ASK1 (Cho et al., 2003), we investigated the possible effect of siRNA-mediated CIIA depletion on ASK1 activity in NSC34 cells expressing human SOD1(WT) or SOD1(G93A). SOD1(G93A) increased the kinase activities of ASK1 and its downstream kinase, p38 MAPK, compared to those of the cells expressing SOD1(WT) (Figure 2A). These results thus suggested that CIIA prevents the SOD1(G93A)-induced stimulation of ASK1 and p38 MAPK. It is noteworthy that CIIA did not affect ROS generation induced by SOD1(G93A) (Figure 2B).

Given that CIIA is able to physically interact with ASK1 (Cho et al., 2003), we examined the CIIA-ASK1 interaction in NSC34 cells expressing either SOD1(WT) or SOD1(G93A). Coimmunoprecipitation analysis revealed that CIIA physically associated with ASK1 in NSC34 cells expressing SOD1(WT), and this association of the CIIA-ASK1 complex was reduced in the cells expressing SOD1(G93A) (Figure 2C, lower panel). These results suggest that SOD1(G93A) promotes the dissociation of CIIA from ASK1, thereby facilitating ASK1 activation. We also examined a possible effect of CIIA on TRAF2-ASK1 interaction, because the recruitment of TRAF2 to ASK1 is an integral part of the mechanism for ROS-induced ASK1 activation (Fujino et al., 2007). SOD1(G93A) induced the binding of TRAF2 to ASK1 in NSC34 cells and this binding was potentiated in those expressing a CIIA siRNA (Figure 2C, upper panel), suggesting that CIIA suppresses the SOD1(G93A)-induced TRAF2-ASK1 interaction.

### CIIA inhibits SOD1(G93A)-induced impairment of mitochondria homeostasis and caspase activation

**Figure 3:**
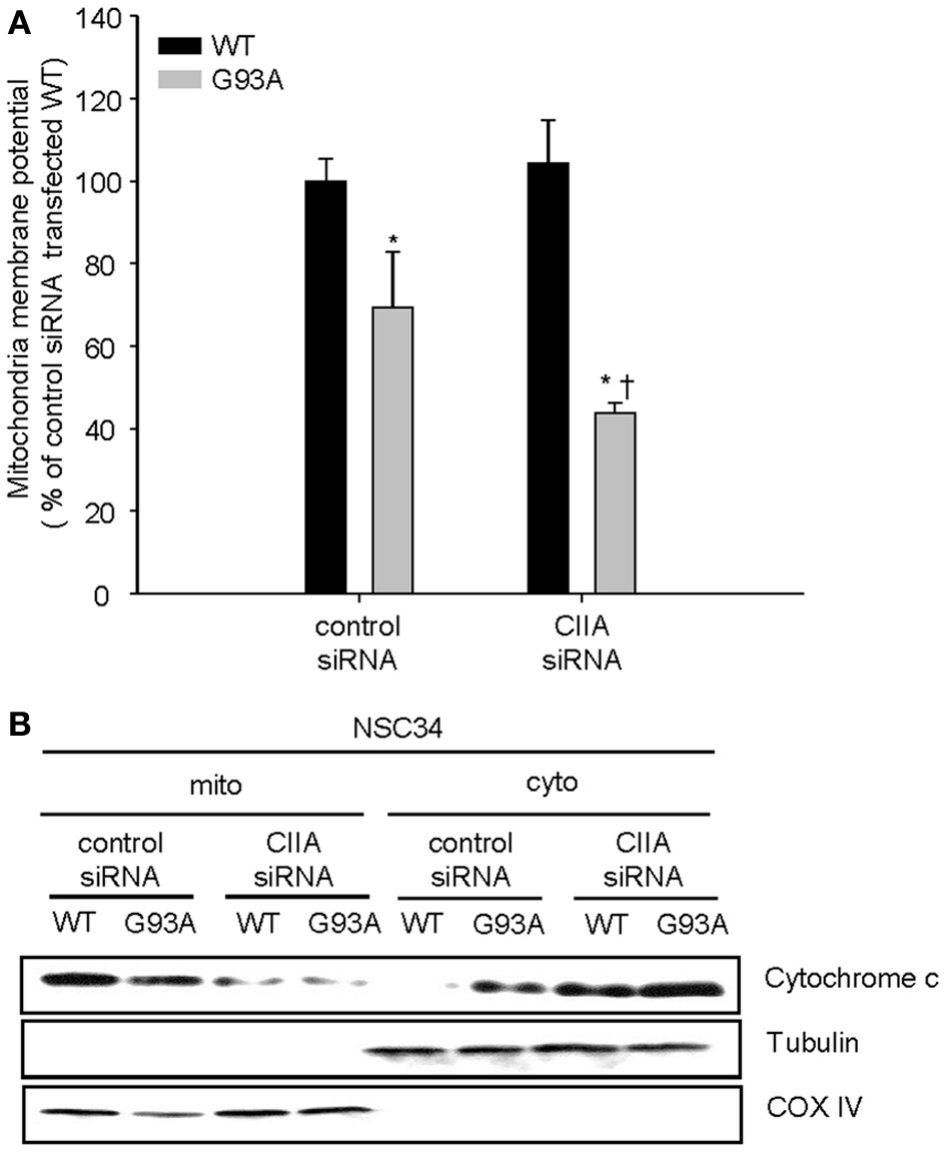
**CIIA inhibits SOD1(G93A)-induced reduction of mitochondria membrane potential (Δψ_m_) and the release of cytochrome c**. NSC34 cells were stably transfected with plasmid vectors encoding Flag-tagged SOD1(WT) or SOD1(G93A) along with vectors for GFP or CIIA siRNA. **(A)** mitochondria membrane potential (Δψ_m_) was analyzed by measuring the fluorescence intensity of tetramethyl rhodamine methyl ester (TMRM, a mitochondria potential sensor). Quantitative data are mean ± s.e.m. from three independent experiments. ^*^P < 0.05 vs. WT; ^†^*P* < 0.05 vs. G93A. **(B)** Cell lysates were subjected to the subcellular fractionation to obtain the mitochondrial (mito) and the cytosolic (cyto) fractions. Each fraction was subjected to immunoblot analysis with antibodies to cytochrome c, to α-tubulin (cytosolic marker), or to COX IV (mitochondrial marker).

**Figure 4:**
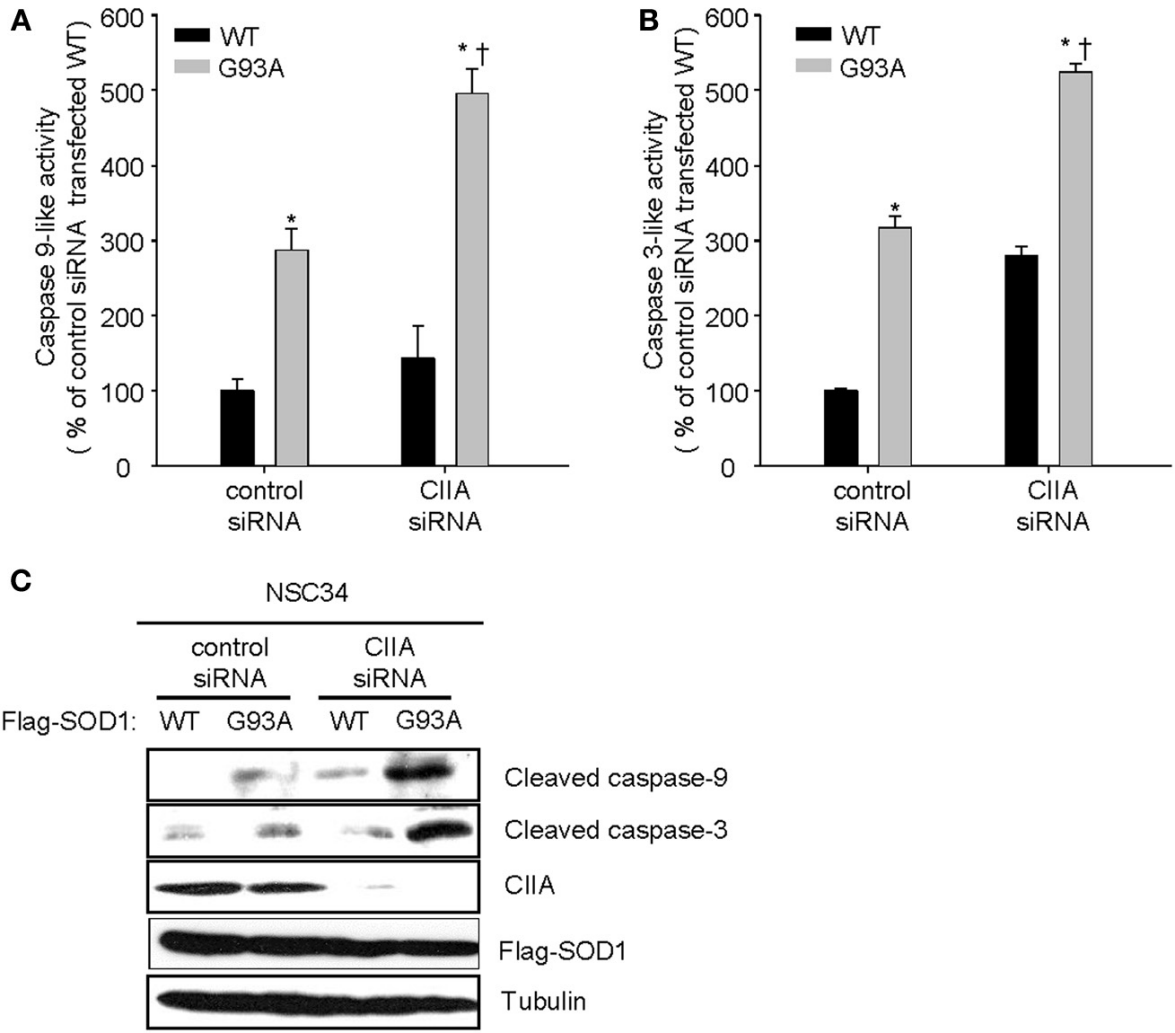
**CIIA inhibits SOD1(G93A)-induced activation of caspase-9 and -3**. NSC34 cells were stably transfected with plasmid vectors encoding Flag-tagged SOD1(WT) or SOD1(G93A) along with vectors for control or CIIA siRNA. (A,B) The cells were incubated with Ac-LEHD-amc **(A)** or Ac-DEVD-amc **(B)**, and then assayed for caspae-9-like **(A)** or caspae-3-like **(B)** activities, respectively, by fluorometry. Quantitative data are mean ± s.e.m. from three independent experiments. ^*^P < 0.05 vs. WT; ^†^*P* < 0.05 vs. G93A. **(C)** Cell lysates were subjected to immunoblot analysis with antibodies to cleaved caspase-9, to cleaved caspase-3, to CIIA, to Flag, or to α-tubulin.

Previously, we reported that the ASK1-p38 signaling pathway mediates the SOD1(G93A)-induced activation of caspase-9 and -3 (Lee et al., 2013). We, therefore, decided to examine a possible effect of CIIA on the mitochondrial intrinsic pathway for apoptosis involving reduction of mitochondrial membrane potential (Δψ_m_), release of cytochrome c from mitochondria, and activation of caspase-9 and -3. SOD1(G93A) reduced Δψ_m_ in NSC34 cells, compared to the cells expressing SOD1(WT) (Figure 3A). Furthermore, depletion of CIIA expression by RNAi promoted the reducing effect of SOD1(G93A) on Δψ_m_ in the cells expressing SOD1(G93A) (Figure 3A). In addition, SOD1(G93A) induced the release of cytochrome c from the mitochondria to the cytoplasm in NSC34 cells and this effect of SOD1(G93A) was enhanced by siRNA-mediated CIIA depletion (Figure 3B). We also examined the effect of CIIA on SOD1(G93A)-induced activation of caspase-9 and -3. SOD1(G93A) induced the increase in the enzymatic activities of caspase-9 (Figure 4A) and -3 (Figure 4B) as well as cleavage (activation) of the caspases (Figure 4C). The RNAi-mediated depletion of CIIA potentiated the effects of SOD1(G93A) on caspase-9 and -3 (Figures 4A–C). Collectively, these results suggested that CIIA negatively regulates the effects of SOD1(G93A) on reduction of Δψ_m_, release of cytochrome c from the mitochondria into the cytoplasm, and activation of caspase-9 and -3.

Previous studies using SOD1^G93A^/ASK1^−/−^ mice have demonstrated that ALS-linked mutant SOD1 induces ER stress and that ASK1 mediates neuronal death induced by SOD1 mutant-induced ER stress (Nishitoh et al., 2008). In this study, we have shown that CIIA physically associates with ASK1 in the cells expressing SOD1(WT), thereby interfering TRAF2-ASK1 interaction, which is a part of mechanism for ASK1 activation. In contrast, SOD1(G93A) induces the release of ASK1 from CIIA and promotes the association of ASK1 with TRAF2, which leads to ASK1 activation. SOD1(G93A)-induced ASK1 activation results in the stimulation of p38 MAPK and impaired mitochondrial homeostasis as well as the activation of caspase-9 and -3, all of which contribute to apoptotic cell death. Depletion of CIIA expression by RNAi potentiated these effects of SOD1(G93A). In relation to these results, our study is under way to investigate whether CIIA overexpression may improve the severity of disease manifestations in the fALS model mice. It would be noteworthy that overexpressed CIIA has been shown to prevent apoptotic cell death mediated by ASK1 activation (Cho et al., 2003). Taken together, these results suggest that CIIA negatively regulates SOD1(G93A)-induced cytotoxicity through inhibiting ASK1. Our findings thus implicate CIIA as a potential modulator of ALS-associated neurotoxicity.

## Materials and methods

### Cell culture and DNA transfection

NSC34 motor neuron–like cells and Neuro2a (N2a) cells were cultured under a humidified atmosphere of 5% CO_2_ at 37°C in Dulbecco's modified Eagle's medium (Gibco, Carlsbad, CA) supplemented with 10% heat-inactivated fetal bovine serum (Gibco). For DNA transfection, cells were plated on glass coverslips in six-well culture dishes or in a 100-mm dish, grown for 24 h, and transfected for 48 h with appropriate vectors with the use of polyethyleneimine (Sigma-Aldrich).

### RNA interference

For depletion of CIIA, NSC34 cells were transfected with a pSuper-retro vector (Oligoengine) for GFP (control) or CIIA siRNA with the use of RNAiMAX (Invitrogen), and the stably transfected cells were selected with puromycin (3 μg/ml). The target sequences for CIIA and GFP were 5'-AAGGCCTACATCAAGGACTGT-3' and 5'-GGCTACGTCCAGGAGCGCACC-3', respectively. For transient transfection of siRNA oligonucleotides with RNAiMAX (Invitrogen), we used GFP (control) siRNA (5' GCTGGAGTACAACTACAACAGCCACAACG-3') and CIIA siRNA (5'-CCTGGGAACAAGCCGGAGCTGTATGAGGA-3').

### Immune complex kinase assay

Immune complex kinase assays were performed as described (Park et al., 2001). GST fusion proteins of MAP kinase kinase 6(K82A) [MKK6(K82A)] and activating transcription factor 2 (ATF2) were used as substrates specific for ASK1 and p38, respectively.

### Co-immunoprecipitation analysis

Cell lysates were incubated for 3 h at 4°C with appropriate antibodies and then for 1 h in additional presence of protein G-conjugated agarose. The resulting precipitates were subjected to immunoblot analysis with the indicated antibodies.

### Preparation of the mitochondrial and cytosolic fractions

NSC34 cells were washed twice with cold PBS and harvested in a cytosolic lysis buffer [250 mM sucrose, 137 mM NaCl, 4.3 mM Na_2_HPO_4_, 1.4 mM KH_2_PO_4_ (pH 7.2), 70 μg/ml digitonin, 0.1 mM phenylmethylsulfonyl fluoride, 1 μg/ml aprotinin, and 1 μg/ml leupeptin] for 30 min on ice. The harvests were subjected to centrifugation for 5 min at 600 × g and the resulting pellets were indicated as the nuclear fractions. The supernatants were centrifuged at 10,000 × g for 10 min and the resulting pellets were designated as the mitochondrial faction and resuspended with a mitochondria lysis buffer [50 mM Tris-HCl (pH 7.4), 150 mM NaCl, 2 mM EDTA, 2 mM EGTA, 0.2% (v/v) Triton X-100, 0.3% NP-40, 0.1 mM phenylmethylsulfonyl fluoride, 1 μg/ml aprotinin, and 1 μg/ml leupeptin]. The supernatants were collected and re-centrifuged at 20,000 × g for 20 min to yield the cytosolic fraction. To verify the fractionation, each fraction was subjected to immunoblot analysis with antibodies to α-tubulin (a cytosolic marker) and COX IV (a mitochondria marker).

### Apoptosis assay

N2a cells were trypsinized, stained with annexin V-FITC and propidium iodide (PI), and analyzed by flow cytometry (FacsCalibur, Becton-Dickinson) with CellQuest software (BD Bioscience) for the detection of apoptotic cells (annexin V-positive and PI-negative). NSC34 cells were fixed, permeabilized, and incubated with TUNEL reaction mixture (Roche, Penzberg, Germany). TUNEL-positive cells were analyzed for apoptosis under Olympus BX53 fluorescence microscope. Data were expressed as the percentage of TUNEL-positive cells.

### Measurement of intracellular ROS production

Intracellular ROS generation was analyzed by dichlorofluorescin (DCF) assay as described (Lee et al., 2013). All data were normalized with respect to the fluorescence intensity of oxidized DCDHF in SOD1(WT)-expressing cells stably transfected with GFP (control) siRNA.

### Measurement of mitochondria membrane potential (Δψ_M_)

NSC34 cells were incubated for 30 min with tetramethylrhodamine methyl ester (TMRM, Invitrogen) at 37°C in the dark. The intensity of TMRM fluorescence was analyzed with FL-600 microplate fluorescence reader (excitation, 360 nm; emission, 460 nm). All data were normalized with respect to the fluorescence intensity of the SOD1(WT)-expressing cells stably transfected with GFP (control) siRNA.

### Caspase assay

NSC34 cells were incubated with 20 μM of fluorogenic substrates (Ac-LEHD-amc and Ac-DEVD-amc) in an assay buffer (50 mM Tris–HCl, pH 7.4, 4 mM DTT, 2 mM EDTA, 10% glycerol, and 0.1% Triton X-100) for 1 h at 37°C in the dark. Ac-LEHD-amc and Ac-DEVD-amc are the fluorogenic substrates specific for caspase-9 and caspase-3, respectively. The relative fluorescence of the cleaved substrate was monitored with FL-600 microplate fluorescence reader (excitation, 360 nm; emission, 460 nm). All data were normalized with respect to the monitored fluorescence in SOD1(WT)-expressing cells stably transfected with GFP (control) siRNA.

### Statistical analysis

Data are expressed as mean ± s.e.m. Comparisons among more than two groups were conducted with analysis of variance (ANOVA) followed by the Student Newman-Kelus test. All analyses were conducted with SPSS version 12.0 software. A *P*-value of < 0.05 was considered statistically significant.

### Conflict of interest statement

The authors declare that the research was conducted in the absence of any commercial or financial relationships that could be construed as a potential conflict of interest.
