# Supplementary material for: CIIA prevents SOD1(G93A)-induced cytotoxicity by blocking ASK1-mediated signaling
Source: Front Cell Neurosci. 2014 Jun 26;8:179. doi: 10.3389/fncel.2014.00179 (PMC4071562; doi:10.3389/fncel.2014.00179)
Supplement: Supplementary file 1 [file DataSheet1.DOCX]

***Supplementary Material***

**CIIA functions as a survival factor against SOD1(G93A)-induced cytotoxicity by inhibiting ASK1 activity**

Jae Keun Lee^1^, Sang Gil Hwang^1^, Jin Hee Shin^2^, Jaekyung Shim^3^, and Eui-Ju Choi^1,*^

^1^Laboratory of Cell Death and Human Diseases, Department of Life Sciences, School of Life Sciences and Biotechnology, Korea University, Seoul 136-701, South Korea; ^2^Department of Health Sciences and Technology, Samsung Advanced Institute for Health Sciences and Technology, Sungkyunkwan University, Seoul 135-710, South Korea; ^3^Department of Molecular Biology, Sejong University, Seoul, 143-747, Korea

*** Correspondence:** Correspondence should be addressed to EJC, Laboratory of Cell Death and Human Diseases, Department of Life Sciences, School of Life Sciences & Biotechnology, Korea University, Seoul 136-701, Republic of Korea. Telephone: +82-2-3290-3446, Fax: +82-2-3290-4741, e-mail: ejchoi@ korea.ac.kr

**Supplementary Figures**


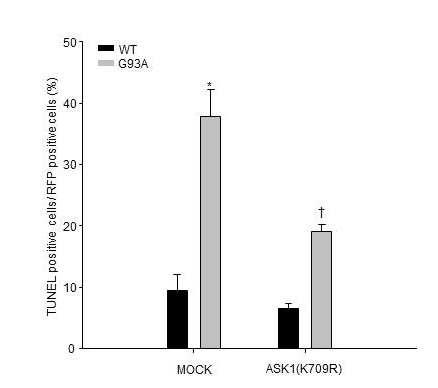


**Supplementary Figure 1. ASK1 mediates SOD1(G93A)-induced cytotoxicity.** NSC34 cells expressing Flag-tagged SOD1(WT) or SOD1(G93A) were transfected with plasmid vector for RFP alone (MOCK) or together with a vector for ASK1 (K709R). The cells were then fixed and examined for apoptosis by TUNEL assay. Quantitative data are means ± SEM. *, *P* < 0.05 versus WT; †, *P* < 0.05 versus G93A.

**
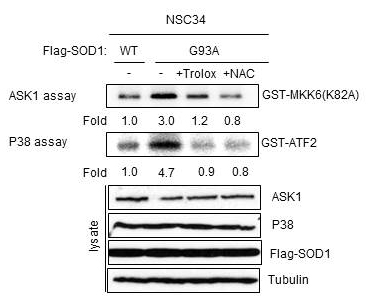
**

**Supplementary Figure 2. SOD1(G93A) induces activation of ASK1 and p38.** NSC34 cells expressing Flag-tagged human SOD1(WT) or SOD1(G93A) were incubated in the absence or presence of 100 μM Trolox or 3mM NAC for 12 h, and then subjected to immune complex kinase assays for ASK1 and p38 kinase activities.
